# Low level of physical activity in women with rheumatoid arthritis is associated with cardiovascular risk factors but not with body fat mass - a cross sectional study

**DOI:** 10.1186/1471-2474-12-13

**Published:** 2011-01-14

**Authors:** Ann-Charlotte Elkan, Niclas Håkansson, Johan Frostegård, Ingiäld Hafström

**Affiliations:** 1 Department of Rheumatology, Karolinska Institutet at Karolinska University Hospital Huddinge, 141 86 Stockholm, Sweden; 2 The National Institute of Environmental Medicine, Division of Nutritional Epidemiology, Karolinska Institutet, 171 77 Stockholm, Sweden; 3 Department of Medicine, Karolinska Institutet at Karolinska University Hospital Huddinge, 141 86 Stockholm, Sweden

## Abstract

**Background:**

As many patients with rheumatoid arthritis (RA) have increased fat mass (FM) and increased frequency of cardiovascular diseases we evaluated if total physical activity (MET-hours) had impact on body composition and cardiovascular risk factors in women with RA.

**Methods:**

Sixty-one out-ward RA women, 60.8 (57.3-64.4) years, answered a self-administered questionnaire, to estimate total daily physical activity during the previous year. Physical activity level was given as metabolic equivalents (MET) × h/day. Diet content was assessed by a food frequency questionnaire and body composition by whole-body dual-energy X-ray absorptiometry. Blood lipids and antibodies against phosphorylcholine (anti-PC) were determined.

**Results:**

Forty-one percent of the women had BMI > 25, 6% were centrally obese and 80% had FM% > 30%. The median (IQR) total physical activity was 40.0 (37.4-47.7), i.e. the same activity level as healthy Swedish women in the same age. Total physical activity did not significantly correlate with disease activity, BMI or FM%. Disease activity, BMI and FM% did not differ between those in the lowest quartile of total physical activity and those in the highest quartile. However, the women in the lowest quartile of physical activity had lower HDL (p = 0.05), Apo A1 (p = 0.005) and atheroprotective natural anti-PC (p = 0.016) and higher levels of insulin (p = 0.05) and higher frequency of insulin resistance than those in the highest quartile. Women in the lowest quartile consumed larger quantities of saturated fatty acids than those in the highest quartile (p = 0.042), which was associated with high oxidized low-density lipoprotein (oxLDL).

**Conclusion:**

This cross sectional study demonstrated that RA women with fairly low disease activity, good functional capacity, high FM and high frequency of central obesity had the same total physical activity level as healthy Swedish women in the same age. The amount of total physical activity was not associated with functional capacity or body composition. However, low total physical activity was associated with dyslipidemia, insulin resistance, low levels of atheroprotective anti-PC and consumption of saturated fatty acids, which is of interest in the context of increased frequency of cardiovascular disease in RA.

## Background

Rheumatoid arthritis (RA) is a chronic inflammatory disease, which may lead to joint destruction, loss of function and increased energy expenditure [1]. Furthermore the disease is often associated with an increase in body fat mass (FM) [2] as well as with reduced life expectancy, mainly due to cardiovascular disease (CVD) [3].

The increase of FM is suggested to be a consequence of the inflammatory process as well as of reduced physical activity [4] as patients with RA have reduced level of physical activity compared to the general population [5], but such a relationship has not been proven.

The increase of CVD is suggested to be related to the effects of the chronic inflammation on the vascular endothelium and to dysregulation of lipid metabolism. Growing evidence points to inflammation in RA being associated with a worsening of the pro-atherogenic lipid profile [6], present already early in the disease [7]. Furthermore patients with RA who are physically inactive have significantly worse CVD risk profile compared with physically active patients [8]. However, the impact of physical activity on FM and risk factors for CVD, such as lipids, is poorly studied in RA.

The aim of this study was to evaluate if total physical activity was associated with body composition, especially FM, and cardiovascular risk factors, such as the atheroprotective anti-bodies against phosphorylcholine (anti-PC), oxidized low-density lipoprotein (oxLDL) and the apolipoprotein profile, in women with RA.

## Methods

### Patients

Sixty-one consecutive out-ward women with RA [9] aged 18-≤ 80 years and with disease duration of ≥ 1 year, at the Rheumatology Department Karolinska University Hospital, Huddinge were included. Exclusion criteria were: current malignancy, severe heart failure [10], severe renal failure (glomerular filtration rate (GFR) < 20 ml/min), chronic obstructive lung disease with emphysema, earlier gastric ulcer or intestinal surgery and known eating disorder.

The study was approved by the Ethics committee at Karolinska Institute, Stockholm, Sweden, reference number 2006/593-31/2, and was performed in accordance with the Helsinki declaration. All patients gave informed consent to participate in the study.

### Disease activity and function

The Disease Activity Score including 28 joints (DAS28) was used, evaluating the number of swollen joints, number of tender joints, the patients' global assessment of health measured on a visual analogue scale (VAS, range 0-100 mm), and erythrocyte sedimentation rate (ESR). The DAS28 score ranges from 0-10, and 2.6-3.2 indicates low disease activity, > 3.2- ≤ 5.1 moderate and > 5.1 high disease activity [11]. Functional status was analyzed using the Swedish version of the Stanford Health Assessment Questionnaire (HAQ), measuring capacity to perform activities of daily living [12]. The HAQ score ranges from 0 to 3, where a higher score indicates a higher degree of disability.

### Physical activity assessment

A short self-administered version of the international physical activity (IPAQ) questionnaire was used to assess average total physical activity during the previous year [13]. It consists of 5 dimensions of physical activity; household work, work/occupation related, walking/bicycling, exercise, and leisure time activities as well as an open question about number of sleeping hours per day. The intensity of activities was defined in multiples of the metabolic equivalents (MET, kcal/kg/h) of sitting quietly (resting metabolic rate) for 1 h [14] and the median MET values were based on specific activities within corresponding categories [15]. The self-reported time was adjusted to 24 h. and multiplied by the intensity factor of 2 MET, corresponding to the average intensity of light activities [16].

### Dietary assessment

Self-administered food-frequency items in the questionnaire (FFQ) asked the patients to report usual frequency of consumption of 88 food items and beverages over the previous year [17]. The nutrient calculations were carried out using nutrient composition values from the Swedish National Food Administration data [18]. The intake of nutrients was computed by multiplying the frequency of consumption of each food item by the nutrient content of the specified portions.

### Biochemical measures

Venous blood samples were drawn between 07:30 and 10:00 after an overnight fast. C-reactive protein (CRP), ESR, plasma glucose, total cholesterol, low-density lipoprotein (LDL) and high-density and lipoprotein (HDL) were determined by standard laboratory methods with commercial kits. Concentrations of total cholesterol > 5.0 mmol/L, LDL ≥ 3.0 mmol/L, HDL < 1.3 mmol/L were considered pathologic.

Apolipoprotein A1 (apoA1) and B (apoB) were determined by Synchrone LX from Beckman AB by turbidimetry. Oxidized LDL (oxLDL) was determined by use of a commercial kit (Mercodia, Uppsala, Sweden) and antibodies against phosphorylcholine (anti-PC) by use of a commercial kit (Athera CVDefine™, Stockholm, Sweden) as described by the manufacturers. At the present time there are no reference values for oxLDL or anti-PC.

### Body composition

*Body mass index (BMI) *was calculated from weight/height^2 ^(kg/m^2^). BMI values < 18.5kg/m^2 ^are considered underweight, between 18.5 - 24.9 as normal, 25-29.9 as overweight and values greater than 30 indicate obesity [19].

*Waist circumference (WC) *was measured to the nearest 0.5 cm midway between the iliac crest and the lower rib margin. According to the International Diabetes Federation (IDF) a waist circumference value less than 80 cm indicate low risk of type 2 diabetes, coronary heart disease or hypertension [20].

*Body composition *was measured with total body dual-energy x-ray absorptiometry (DXA) (GE-Lunar Prodigy, software enCore 2006, version 10, 20,105, Madison, USA). The precision of soft tissue analysis for a Lunar Prodigy is 1% for fat free mass (FFM) and 2% for FM [21]. FFM and FM were expressed in absolute kilograms and FM as well as percentage of total mass. The reference value for FM% is for women 20-30% [22]. FM index (FMI kg/m^2^) was also calculated.

## Statistics

Data were presented as mean (confidence interval) or median (interquartile range) depending on whether the data were normally distributed or not. Differences between patient groups were assessed using the Student t test or Mann-Whitney U test, depending on the distribution of the analyzed variable. Spearman rank correlation was used for univariate analysis. *P *values < 0.05 were considered significant. The statistical analysis program Statistica 8 (Stat Soft Scandinavia AB, Uppsala, Sweden), was used for statistical analysis.

## Results

*Table *1 presents demographic, disease-related and anthropometric characteristics for the patients. The patients had a fairly low disease activity according to DAS28 and a good functional ability as registered by HAQ. All patients except two were treated with disease-modifying drugs (DMARDs).

**Table 1:** Demographic and anthropometric characteristics for the RA women.

	RA women all N = 61	MET-hours 1^st ^quartile N = 14	MET-hours 4^th ^quartile N = 14	P-value
Age (years)	60.8 (57.3-64.4)	60.0 (57.0-65.0)*	66.0 (63.0-76.0)*	0.09
Ever smoker, N, (%)	39 (64)	11 (79)	7 (50)	0.29
RA duration*	6.0 (2.0-15.0)	4.5 (2.0-10.0)	5.5 (2.0-14.0)	0.68
DAS28	3.3 (3.0-3.6)	3.4 (2.8-4.1)	3.1 (2.3-3.9)	0.32
ESR (mm/h)*	16.0 (9.0-29.0)	18.0 (13.0-21.0)	10.0 (5.0-27.0)	0.23
HAQ *	0.5 (0.13-1.13)	0.82 (0.25-1.0)	0.63 (0.13-1.13)	0.64
Patients on glucocorticoids, N, (%)	17 (28)	4 (31)	3 (20)	0.26
Glucocorticoids, mg*	5.0 (2.50-5.0)	3.12 (1.78-4.37)	3.9 (2.5-5.0)	0.54
Patients on anti-TNF, N, (%)	15 (25)	3 (23)	4 (27)	0.60
BMI (kg/m^2^)*	24.2 (21.6-26.6)	25.9 (23.6-29.5)	22.8 (20.4-26.5)	0.20
Waist (cm)*	83.0 (75.0-91.5)	87.2 (80.0-99.5)	79.0 (72.0-90.0)	0.11
Fat mass (%)	37.8 (35.7-39.9)	41.0 (36.1-45.9)	35.1 (31.1-39.0)	0.30
FMI (kg/m^2^)*	8.8 (6.4-11.4)	11.5 (8.4-14.7)	6.6 (6.3-11.1)	0.14
FFMI (kg/m^2^)*	15.3 (14.6-16.2)	17.5(14.7-17.1)	15.3 (14.7-16.7)	0.52
MET-hours*	40.0 (37.4-44.7)	35.9 (35.4-37.1)	45.8 (45.2-49.2)	**< 0.001**

The RA women are separated into the first and fourth quartiles of total physical activity, assessed as MET (= metabolic equivalents) hours.

Data is presented as mean (CI) for normally distributed variables and as median (inter-quartile range) for non-parametric variables, * = median, N = numbers, % = percentage. DAS28 = Disease Activity Score including 28 joints, ESR = erythrocyte sedimentation rate, HAQ = Health Assessment Questionnaire, anti-TNF = Anti-tumour necrosis factor therapy, BMI = body mass index, FMI = fat mass index, FFMI= fat free mass index.

Forty-one percent of the women had BMI > 25, and 57% were centrally obese (waist circumference > 80 cm). Eighty percent had FM% > 30%.

The total physical activity level was median (IQR) 40.0 (37.4-44.7) MET-hours/day, which corresponds to the activity of healthy Swedish women of the same age [13]. However in 21% the physical activity was low.

### Physical activity in relation to patient characteristics and disease related measures

Total physical activity did not significantly associate with DAS28 or HAQ-score. When dividing total physical activity into predefined activity time categories it was evident that women with DAS28 ≥ 3.2 did less walking than women with DAS28 ≤ 3.2 (p = 0.009). There were no significant differences in the other categories of physical activity. As shown in *table *1, patients in the lowest quartile of total physical activity did not differ in age, disease duration or disease activity and severity from those in the highest quartile of physical activity.

### Total physical activity and body composition

Total physical activity did not significantly correlate with BMI, FM% or FMI. Patients in the lowest quartile of total physical activity did not differ in body composition or waist circumference from those in the highest quartile, *table *1.

### Total physical activity, blood lipids, anti-PC and insulin resistance

Fifty-three percent of the women had cholesterol > 5.0 mmol/l and 51% had high LDL levels. The women in the lowest quartile of total physical activity had significantly lower values of HDL (p = 0.05), Apo A1 (p = 0.005), the atheroprotective natural anti-PC (p = 0.016) and higher levels of insulin (p = 0.05) than those in the highest quartile, *table *2. Insulin resistance was present in 30% of the women in the lowest quartile and in 13% in the highest.

**Table 2:** Lipids and insulin levels of the RA women and groups of total physical activity

	MET-hours 1^st ^quartile	MET-hours 4^th ^quartile	p-value
Cholesterol, mmol/L	4.9 (4.6-5.7)	5.7 (4.6-6.3)	0.12
HDL, mmol/L	1.6 (1.4-2.1)	2.1 (1.7-2.5)	**0.004**
LDL, mmol/L	2.7 (2.2-3.2)	2.9 (2.4-3.4)	0.62
oxLDL, U/L	57.0 (45.4-68.6)	61.9 (48.7-75.1)	0.60
Apo A1, g/L	1.44 (1.32-1.51)	1.78 (1.59-1.9)	**0.005**
Apo B, g/L	0.91 (0.76-0.98)	0.86 (0.70-1.01	0.61
ApoB/Apo A1, g/L	0.61 (0.50-0.71)	0.51 (0.42-0.61)	0.15
Anti-PC, U/mL	36.8 (20.7-45.0)	45.3 (39.3-70.1)	**0.016**
Insulin, pmol/L	43.0 (27.1-112.1)	31.4 (22.9-47.4)	**0.05**

Data is presented as median (inter-quartile range), LDL = low-density

lipoprotein, HDL = high-density lipoprotein, Apo A1 = apolipoprotein A1,

apoB = apolipoprotein B, OxLDL= oxidized low-density lipoprotein,

anti-PC = antibodies against phosphorylcholine.

### Total physical activity and dietary intake

Compared with the Swedish Food Recommendations for individuals with low physical activity [23] the patients reported low intake of total energy, mean (CI) 1668 (1537-1799) kcal/day.

There were no significant differences in total energy intake in the different quartiles of total physical activity. However, the patients in the lowest quartile consumed significantly more saturated fatty acids (SFAs) than those in the highest quartile (p = 0.042).

In univariate correlation analyses there was a significant positive correlation between intake of SFAs and oxLDL (r = 0.26, p = 0.047) and trend wise for Apo B (r = 0.24, p = 0.057). A trend for a negative correlation between intake of SFAs and HDL (r = 0.25, p = 0.055) was also found.

## Discussion

This cross sectional study demonstrates that woman with RA and fairly well controlled disease and good functional capacity were in general obese. In average they had the same total physical activity level as healthy Swedish women in the same age [13], albeit in 21% the physical activity was low. Despite low total physical activity this subgroup of patients did not differ in content of fat mass from those with the highest level of total physical activity, but several risk factors for CVD were present.

The presence of increased FM in patients with RA, also reported earlier, has been suggested to partly be due to low physical activity [24]. In the present study, however, the amount of reported total physical activity was not associated with body composition. Neither in patients in the lowest quartile of total physical activity was FM, BMI or waist circumference different from those in the highest quartile. This independence is in contrast to the finding by Stavropoulos-Kalinoglou et al. who reported that higher levels of physical activity were associated with lower BMI and FM in patients with RA [4]. However, there are several discrepancies between their study and the present. Thus in that study, which also included men, the patients were more handicapped as measured by HAQ and had lower FM% compared with ours. Furthermore physical activity was measured only over the previous week, as compared with one year in the present study. Lastly body composition was assessed by bioelectrical impedance analysis (BIA) with Tanita BC-418, which systematically underestimates FM in patients with RA [25].

It is probable that the level of total physical activity in the present patients was not high enough to reduce FM. Thus, still higher activity level seems to be needed, as progressive resistance training twice a week for 24 weeks ameliorated the body composition among RA patients with well-controlled disease [26]

However, although a subset of the present patients were physically inactive; the patients had, in average, the same level of total physical activity as healthy Swedish women in the same age. This is in contrast to the general conception that patients with RA are less active than healthy persons. Patients with RA are a heterogeneous group and as reported in a large multicenter study physical inactivity was more prevalent in patients with poor functional capacity and higher disease activity than those with better clinical status [27].

These data are in line with our results. The RA women in our study, with higher disease activity, did less walking than women with lower disease activity. Thus walking might induce anti-inflammatory responses known to be a result of exercise [28]. There were no significant differences in the other activity time categories. This is also in line with reports from Mancuso et al who have shown that RA patients have less weekly total energy expenditure from physical activity than healthy controls, and this is primarily due to less walking [29]

Of course, also the accuracy of self-reported physical activity can be discussed as that may be subject to reporting bias with both under-and over-estimation of physical activity. However, the present questionnaire has been tested against objective measures among Swedish women and is considered to be valid [13].

About one half of the present RA women displayed dyslipidemia, [6,7,30], and in the lowest quartile of total physical activity they had significantly lower levels of HDL, Apo A1, anti-PC and higher levels of insulin than those in the highest quartile. These findings in patients with low physical activity are all pro-atherogenic and very interesting in the context of cardiovascular disease in RA.

Anti-PC are natural antibodies, which are suggested to play a role in atherogenesis and chronic inflammation. Phosphorylcholine (PC) is a major ligand in oxLDL, exposed on platelet activating factor (PAF)-like phospholipids, which promote inflammation [31]. Levels of anti-PC are inversely associated with development of atherosclerosis in patients with established hypertension [32]. Further, low levels of anti-PC are associated with an increased risk of development of CVD [33].

In RA anti- PC has not been studied in relation to CVD, but we have recently shown that the levels of anti-PC in serum increased in RA patients when changing from a normal western diet to a gluten-free vegan diet [34] and that patients on a mediterranean like diet had higher levels of anti-PC than those on a normal western diet [35].

The total dietary intake did not differ between patients in the lowest and those in the highest quartile of total physical activity, in line with the earlier reported non-association between total dietary intake and body composition [4]. However, the patients with the lowest level of total physical activity consumed significantly more SFAs, which could be a reflection of a less healthy lifestyle. The intake of SFAs also correlated positively with levels oxLDL, which is interesting as LDL oxidation probably has an important role in the pathogenesis of atherosclerosis [36].

Low physical activity and high dietary intake of SFA were thus associated with lipid cardiovascular risk factors differently; low physical activity with low HDL, Apo A1, anti-PC as well as high insulin levels, and high dietary intake of SFA with high oxLDL.

The impact of low long-term physical activity on risk factors for CVD in RA has not been studied earlier. That this interaction has clinical implications is though shown in the general population as Swedish women with low total physical activity (≤ 35 MET h/day) have a 3.22 times increased mortality compared with the active women (> 50 MET h/day) [37].

Higher habitual intakes of SFA are independently associated with increased atherosclerosis [38], consequences that would encourage the physician to recommend the RA patient to decrease the consumption of SFAs in the prevention of cardiovascular diseases.

The limitations of the current study should be acknowledged. The cross-sectional design and small size of our study limits the ability to ascribe causal relationships to the associations detected. Cross-sectional studies only provide a 'snapshot' of the outcome and the characteristics associated with it at a specific point in time. However, the instruments for analysing physical activity and dietary intake were chosen to meassure the whole previous year.

## Conclusions

In summary this study shows that in relatively obese women with RA low level of total physical activity displayed significantly lower levels of HDL, ApoA1, the atheroprotective anti-PC and significantly higher levels of insulin than in those with higher physical activity. These women also consumed significantly more SFAs than those who were more active, a diet associated with high oxLDL. Despite these findings the extent of physical activity was not associated with the amount of body fat. Thus, physical inactivity seems to have greater impact on risk factors for CVD other than fat mass in patients with RA.

## Competing interests

The authors declare that they have no competing interests.

## Authors' contributions

ACE was responsible for the design of the study, for all measurements, for analysing the data and was responsible for writing the manuscript. NH had the responsibility for analysing the FFQ. JF was responsible for the assays of lipids and anti-PC. IH took active part in the design of the study and has been senior advisor in all parts of the research and manuscript preparation. All authors read and approved the final manuscript.

## Pre-publication history

The pre-publication history for this paper can be accessed here:

http://www.biomedcentral.com/1471-2474/12/13/prepub
